# Factors affecting T2DM patients’ behaviors associated with integrated treatment and prevention services in China

**DOI:** 10.1186/s12939-023-02028-9

**Published:** 2023-10-19

**Authors:** Ran Zhao, Xia Zhang, Sizhe Wang, Nan Zhao, Dianjiang Li, Hong Fan

**Affiliations:** 1 School of Public Health, Nanjing Medical University, 101 Longmian Road, Nanjing, P. R. China; 2 School of Nursing, Nanjing Medical University, 101 Longmian Road, Nanjing, P. R. China

**Keywords:** Behaviors, Integrated treatment and prevention services, Theory of planned behavior, Structural equation model

## Abstract

**Objective:**

To explore the relationship between type 2 diabetes mellitus (T2DM) patients’ attitude, subjective norms (SN), perceived behavioral control (PBC), behavioral intention (BI) and behavior associated with integrated treatment and preventive (ITP) services.

**Methods:**

A convenient sampling method was employed at a community health center in Nanjing, China between January and July 2022. The collected data were processed using Epidata 3.1, SPSS 26.0, and AMOS 24.0. Descriptive statistics and a structural equation model based on the theory of planned behavior (TPB) were used to explore the correlation between the study variables.

**Results:**

430 participants were eventually included, with a response rate of 98.6%. The mean age was 72.50 ± 5.69 years. The TPB model proved to be suitable and explained 41% of the variance in the BI. Attitude (β = 0.289, P < 0.001), SN (β = 0.314, P < 0.001) and PBC (β = 0.261, P < 0.001) were the main predictors of BI, and the SN was the strongest. BI (β = 0.452, P < 0.001) and PBC (β = 0.452, P < 0.001) had similar direct effects on patients’ behavior.

**Conclusion:**

The TPB model explained the behavioral variations associated with ITP services and provided a framework for developing targeted interventions and improving community-based ITP services for T2DM. To encourage patients to engage in desirable behaviors, interventions should focus on modifying patients’ SN towards behavior associated with ITP services by promoting peer pressure and increasing the family’s emphasis on health.

**Supplementary Information:**

The online version contains supplementary material available at 10.1186/s12939-023-02028-9.

## Introduction

### Diabetes in China

According to the International Diabetes Federation, 536.60 million people worldwide had diabetes in 2021 [1]. By 2045, this number is expected to increase to 783.70 million [1]. China is the epicenter of diabetes, with 141 million people suffering from the disease in 2021 [2], and the prevalence (% of population ages 20 to 79) was 10.6% [3]. Type 2 diabetes mellitus (T2DM) progression and its potential complications will not only worsen patients’ quality of life and increase their financial and psychological burden [4], but also bring serious harm to families and society. China’s total spending on diabetes in 2021 was $165.3 billion, ranking second in the world [2]. From 1990 to 2019, diabetes increased at an annual rate of 2.27% per 100,000 disability-adjusted life years (DALYs) in China [5], posing a serious threat to the health of Chinese residents. Diabetes has become one of the major public health problems that need to be solved urgently.

### Integrated treatment and prevention

The fragmentation of the prevention and treatment of T2DM in China’s health service system has resulted in the inability of patients to receive continuous and efficient diabetes health management, which has seriously hindered the improvement and effectiveness of diabetes prevention and treatment [6, 7]. Combining the prevention and treatment of patients with T2DM not only connects healthcare institutions at different levels, provides comprehensive services for patients, and improves the efficiency of service delivery, but also moderately reduces the level of HBA1c in patients with T2DM [8, 9]. In response to the Global Strategy of Human-Oriented Integrated Health Services issued by World Health Organization (WHO) and the improvement of healthy life expectancy in China [10], China has proposed strengthened collaboration between treatment and prevention and promoted the integrated development of chronic disease prevention, treatment, and management [11]. Although integrated treatment and preventive (ITP) services are not explicitly defined, similarities may be drawn with comprehensive care insofar as the concept may be described as a coherent and coordinated collection of services provided to patients through various organizations, professionals and caregivers [12]. Strong evidence supports the view that integrated, patient-centered care for T2DM patients based on a chronic care paradigm ($11,339/QALY) is significantly more affordable than usual care [13].

Previous studies on the prevention and treatment of diabetes mainly focused on the self-care and self-management behaviors of patients. This study incorporates a public health service utilization component on the basis of diabetic patients’ self-management, and gives more prominence to the systematicity and continuity of community diabetes health service, in order to analyze the ability of the patients to manage and control their own glycemia with the help of their own and ambient health services. The implementation of ITP services is still in its early stage, and the utilization of these services remains suboptimal. A cross-sectional study conducted in Shandong, China, revealed that only 75.8%, 74.5%, 61.0%, and 25.8% of patients demonstrated adequate self-management with regards to medication compliance, diet control, physical exercise, and self-monitoring of blood sugar levels, respectively [14]. A mere 15% of patients had undergone tests for diabetic retinopathy and kidney disease in the past year [15]. Moreover, a negligible number of individuals with diabetes followed proper foot care practices [16]. Furthermore, patients tend to primarily use hospital services, resulting in underutilization of primary health-care services [17]. The process of behavioral change is complex and influenced by a multitude of factors such as knowledge, beliefs, attitudes, abilities, motivation, and social support [18]. Therefore, it is essential to analyze the factors affecting patients’ behavior associated with ITP services to better clarify the mechanism underlying the implementation of patients’ behavior, identify areas for improvement, and promote the implementation of ITP services in China for better health outcomes.

In this study, ITP services were defined as a comprehensive, systematic, and continuous patient-centred service that integrates basic medical care and basic public health services based on the theory of continuum of services and health management [19, 20]. According to the health and medical service needs of T2DM patients, this paper integrated medical treatment, prevention, health care and other related services, and conducted an in-depth study on the utilization of ITP services for T2DM from seven perspectives: diet control, exercise, medication compliance, foot care, glucose and blood pressure monitoring, complication screening, and public health service utilization.

### Theoretical framework

The Theory of Planned Behavior (TPB), developed by Icek Ajzen in 1985, is a widely recognized theory that endeavors to predict human behavior based on their intentions [21, 22]. It builds upon the Theory of Rational Behavior and argues that an individual’s behavior is contingent upon their behavioral attitude (BA), subjective norms (SN), perceived behavioral control (PBC), and behavioral intention (BI) toward that behavior [23]. The TPB has been applied to explain a wide range of health-related behaviors, including substance abuse [24], factors influencing smoking behavior [25], diabetes management through a low-sugar diet [26], and healthcare-seeking behavior [27]. There is substantial evidence to support the efficacy of TPB in explaining the processes that influence behavior.

This study adapted the TPB as its theoretical framework and integrated seven dimensions to evaluate patient behavior associated with ITP services. The purpose was to uncover the key factors and inter-factor mechanisms based on TPB, that influence patients’ adoption of behavior associated with ITP services. This will provide a sound theoretical and empirical basis for enhancing patients’ glucose management ability, improving the quality of community ITP services, and informing future community intervention studies.

## Methods

### Study design and participants

Between January and July 2022, a cross-sectional survey was conducted among T2DM patients at the Jiangning District Health Service Center in Nanjing, China. The sample for the study was selected through convenience and comprised of individuals who were permanent residents of Jiangning District and had a health record at a community health service center. Eligible participates were invited to participant in the survey after meeting the criteria of possessing strong communication abilities and being free from mental health concerns.

### Questionnaire design

#### Construct questionnaire items

The questionnaire items were constructed with TPB as the theoretical framework, and the research object, context, time, and behavior were strictly defined before measurement, to ensure that the BA, SN, PBC and BI in the measurement questionnaire all pointed to the same specific behavior. The actual situation of T2DM patients in the community was researched and recorded through a literature review, group discussion and individual in-depth interviews. A pre-investigation was conducted on 30 T2DM patients in a community health service center in Jiangning District, Nanjing. The contents and factors affecting the behavior associated with ITP services of T2DM patients were understood according to the actual literature review and questionnaires, and the questionnaire items of this study were constructed based on the TPB framework combined with expert suggestions.

#### Form an initial questionnaire

This survey was based on the simplified scale of Diabetes Self-management Knowledge, Attitude, and Behavior Assessment Scale (DSKAB-SF) developed by Chinese Center for Disease Control and Prevention, supplemented with three dimensions of SN, PBC and BI, and designed the questionnaire of factors affecting T2DM patients’ behavior associated with ITP services based on TPB. The draft questionnaire was then revised and improved according to expert suggestions. Each sub-questionnaire included related items from basic public health services in addition to self-management of diabetes, and the utilization of ITP services by patients was studied from two perspectives of basic public health and basic medical treatment.

### Data collection

According to literature, Exploration Factor Analysis (EFA) necessitates at least 5–10 times the number of items in the sample [28], in this case, a minimum of 205 participants. The Confirmatory Factor Analysis (CFA) requires at least 200 samples [29]. To accommodate a 5% allowance for invalid questionnaires, the study aimed to recruit a minimum of 425 participants. Through convenience sampling, a total of 436 T2DM patients (N = 436) were selected. Upon exclusion of invalid cases with missing data, 430 questionnaires (n = 430) were finally included, yielding an effective response rate of 98.6%. The sample was further divided into the EFA and CFA groups through random case selection in SPSS, with 209 and 221 individuals assigned to each group respectively.

Initially, leaders of the health service center and relevant departments were contacted in order to clarify the purpose and object of the research and seek to obtain strong support and cooperation from the center. Convenience sampling was used to select T2DM patients who met the inclusion and exclusion criteria in the center as the survey objects. Data collection was conducted through face-to-face interviews by trained team investigators in accordance with relevant regulations. The purpose of the questionnaire was explained at the outset of interviews and guarantees given regarding the confidentiality of the questionnaires. After obtaining the informed consent of patients, respondents were invited to complete a series of questionnaires, including socio-demographic information, disease-related data, and the ITP questionnaire.

### Measures

The evaluations were measured by asking survey respondents to rate using a five-point Likert scale. The results of the Cronbach’s alphas test indicated high internal consistency within each construct, with values exceeding 0.8 for all sections.

#### Behavioral attitude (BA, 6 items)

BA refers to the positive or negative evaluations held by individuals with T2DM towards the adoption of behaviors associated with ITP services [23] (e.g. ‘Do you think it is important for diabetics to take medication according to doctor’s requirements for blood glucose control?’). A higher score indicated a more favorable attitude towards the individual’s practice of actual behaviors.

#### Subjective norms (SN, 6 items)

SN describes the social pressure experienced by patients with T2DM in relation to their adoption of behaviors associated with ITP services. This construct gauges the extent to which individual behavior decisions were influenced by important people, such as doctors, family members and friends (e.g. ‘The influence of the surrounding population on your adherence to the principles of diabetes medication’). A higher score indicating a stronger impact of the perceived influence of others on the patients to take certain behaviors.

#### Perceived behavioral control (PBC, 6 items)

The construct of PBC is employed to assess the T2DM patients’ perceptions of the difficulty in relation to the performance of behaviors associated with ITP services. This construct in this study was used to evaluate an individual’s perceived ability to control their behavior (e.g. ‘their ease of adhering to take medication or/and take insulin injection as prescribed’). With higher scores indicating a stronger sense of control and a lower perception of obstruction in performing the relevant behaviors.

#### Behavioral intention (BI, 6 items)

BI refers to the direction and strength of an individual’s thought process before they make a decision to perform behaviors associated with ITP services (e.g. ‘whether they plan to adhere to a medication regimen or/and insulin injections as prescribed’). The higher the score, the more likely the individual was to adopt and implemented the relevant behaviors.

#### Actual behavior (AB, 17 items)

The construct of AB was assessed through a 17-item subscale in this study (e.g. ‘Have you used a combination of diet, exercise and medication to control your blood sugar during the past six months?’). With higher scores indicating more favorable behavior performance.

### Data analysis

The collected data were processed using Epidata 3.1, SPSS 26.0 (SPSS, Inc., Chicago, IL, USA), and AMOS 24.0 (Amos Development Corporation) as follows.

1. Descriptive statistical analysis, including mean, standard deviation, frequency, and percentage, were performed to characterize the participants.
2. In the EFA, the common factors among all items were extracted using principal component analysis. The Kaiser-Meyer-Olkin (KMO) sample adequacy measure and the Bartlett’s test of Sphericity, were used to evaluate the data’s factorability. The questionnaire was considered to have structural validity if the KMO value was greater than 0.6 and the significance probability of the Chi-square value of the Bartlett’s sphericity test was less than 0.05 [30].
3. CFA was performed to examine the relationship between the items and factors, and a measurement model was constructed between the latent variables and observable indicators. The critical values for evaluating the goodness of fit between the model and data are shown in Table 1 [31–33].
4. A structural equation model (SEM) was developed based on the TPB to identify the interrelationships among latent variables. The parameters were estimated using the maximum likelihood (ML) method.

**Table 1:** Categorical confirmatory factor analysis (N = 221)

Latent Variables	Item	Std.	S.E.	C.R.	P	SMC	CR	AVE	Factor Correlation				
									BA	SN	PBC	BI	Be
**BA**	BA1	0.827				0.684	0.920	0.659	**0.812**				
	BA2	0.832	0.072	14.659	***	0.692							
	BA3	0.770	0.071	13.225	***	0.593							
	BA4	0.840	0.069	14.907	***	0.706							
	BA5	0.806	0.072	14.029	***	0.650							
	BA6	0.793	0.077	13.622	***	0.629							
**SN**	SN1	0.760				0.578	0.901	0.604	0.297	**0.777**			
	SN2	0.795	0.094	12.046	***	0.632							
	SN3	0.802	0.092	12.184	***	0.643							
	SN4	0.735	0.089	11.005	***	0.540							
	SN5	0.815	0.090	12.477	***	0.664							
	SN6	0.753	0.097	11.364	***	0.567							
**PBC**	PBC1	0.795				0.632	0.902	0.605	0.402	0.301	**0.778**		
	PBC2	0.744	0.080	11.854	***	0.554							
	PBC3	0.802	0.078	12.929	***	0.643							
	PBC4	0.762	0.080	12.162	***	0.581							
	PBC5	0.829	0.090	13.532	***	0.687							
	PBC6	0.729	0.087	11.469	***	0.531							
**BI**	BI1	0.800				0.640	0.920	0.659	0.440	0.404	0.497	**0.812**	
	BI2	0.855	0.075	14.515	***	0.731							
	BI3	0.790	0.072	13.044	***	0.624							
	BI4	0.766	0.076	12.434	***	0.587							
	BI5	0.822	0.082	13.668	***	0.676							
	BI6	0.834	0.076	14.112	***	0.696							
**Be**	Be1	0.855				0.731	0.949	0.729	0.675	0.569	0.681	0.633	**0.854**
	Be2	0.849	0.044	16.646	***	0.721							
	Be3	0.905	0.059	18.759	***	0.819							
	Be4	0.738	0.026	13.253	***	0.545							
	Be5	0.895	0.059	18.332	***	0.801							
	Be6	0.894	0.059	18.274	***	0.799							
	Be7	0.827	0.044	15.887	***	0.684							

Note: ***<0.001, Std. (Standardized Factor Loading), S.E. (Standard Error), C.R. (Critical Ratio), SMC (Squared Multiple Correlation), CR (Construct Reliability), AVE (Average Variance Extracted), Be (Behavior analysis entries that packages the measurement items of AB by dimension)

## Results

### Characteristics of participants

The mean age of participants in this study was 72.50 ± 5.69 years, with 71.9% of them being female (Table 2). The average duration of the disease was 10.67 ± 7.26 years. Approximately 50.0% of patients were classified as overweight. 34.7% had only received primary education, and 42.8% were illiterate. Most patients were either married or cohabiting (76.5%), unemployed (94.7%), or had low-incomes (96.7%). 11.6% of respondents lacked health insurance. Although the majority of patients had no family history of diabetes (69.1%), nearly half of the patients required either mono-insulin or combination therapy (48.6%).

**Table 2:** Characteristics of Participants

Variables	
**Sociodemographic data**	
Age, years (mean ± SD*)	72.50 ± 5.69
Gender, N (%)	
*Male*	121 (28.1)
*Female*	309 (71.9)
Education level, N (%)	
*University and above*	5 (1.2)
*Senior high school/technical secondary school*	19 (4.4)
*Junior high school*	73 (17.0)
*Primary school*	149 (34.7)
*Illiteracy*	184 (42.8)
Marriage, N (%)	
*Single #*	101 (23.5)
*Non-single*	329 (76.5)
Occupation, N (%)	
*Employed*	23 (5.3)
*Unemployed*	407 (94.7)
Monthly income, RMB, N (%)	
*< 3000*	415 (96.7)
*3000 ~ 5000*	8 (1.9)
*5000 ~ 8000*	5 (1.2)
*>=10,000*	1 (0.2)
Health insurance, N (%)	
*State medicine*	1 (0.2)
*Urban employee basic medical insurance*	6 (1.4)
*Urban-Rural Resident Basic Medical Insurance*	370 (86.0)
*Commercial insurance*	1 (0.2)
*Other*	2 (0.5)
*None*^*♄*^	50 (11.6)
**Clinical data**	
Disease course^￠^, years, (mean ± SD)	10.67 ± 7.26
Body mass index ^♆,^ kg/m^2^, N (%)	
*< 18.5*	4 (0.9)
*18.5 ~ 24.0*	131 (30.5)
*24.0 ~ 28.0*	215 (50.0)
*>=28.0*	80 (18.6)
Family history ¤, N (%)	
*Yes*	133 (30.9)
*No*	297 (69.1)
Treatment strategies, N (%)	
*Diet and exercise*	3 (0.7)
*Oral hypoglycemic agents*	218 (50.7)
*Insulin monotherapy*	153 (35.6)
*Insulin combined with oral hypoglycemic agents*	56 (13.0)

Note: SD^∗^: standard deviation. Single^#^: including divorced or widowed. None^♄^: without any health insurance. Disease course^￠^: Interval between diagnosis of diabetes and completion of questionnaire. Body mass index ^♆^ (BMI): BMI (24.0 ~ 28.0) meaning overweight. Family history^¤^: Whether a blood relative had diabetes

### Exploratory factor analysis

A comprehensive evaluation of the questionnaire was performed using EFA, which revealed that it possessed a high degree of construct validity, as evidence by Kaiser-Meyer-Olkin (KMO) value of 0.952, and a high significant result (P < 0.001) in Bartlett’s test of sphericity. The factor analysis resulted in the extraction of five principal components, and the items that comprised these factors were found to be aligned with the dimensions intended by the original questionnaire design. The extracted components collectively accounted for 66.4% of the total variability in the data, suggesting that the data set was appropriate for conducting factor analysis (Table 3).

**Table 3:** Results of Exploratory Factor Analysis (N = 209)

Item	Factor				
	1	2	3	4	5
BA1				0.757	
BA2				0.780	
BA3				0.760	
BA4				0.800	
BA5				0.760	
BA6				0.792	
SN1			0.805		
SN2			0.789		
SN3			0.742		
SN4			0.796		
SN5			0.737		
SN6			0.799		
PBC1		0.725			
PBC2		0.802			
PBC3		0.792			
PBC4		0.786			
PBC5		0.759			
PBC6		0.805			
BI1					0.780
BI2					0.718
BI3					0.715
BI4					0.797
BI5					0.741
BI6					0.765
AB1	0.634				
AB2	0.625				
AB3	0.642				
AB4	0.682				
AB5	0.632				
AB6	0.614				
AB7	0.758				
AB8	0.667				
AB9	0.675				
AB10	0.622				
AB11	0.694				
AB12	0.653				
AB13	0.678				
AB14	0.739				
AB15	0.649				
AB16	0.713				
AB17	0.706				
Eigenvalue	9.207	4.542	4.540	4.519	4.414
Variance (%)	22.455	11.079	11.074	11.021	10.766
Accumulative variance (%)	22.455	33.534	44.608	55.629	66.395

Note: BA (Behavioral attitude), SN (Subjective norms), PBC (Perceived behavioral control), BI (Behavioral intention), AB (Actual behavior)

### Confirmatory factor analysis

A CFA test was conducted on 5 factors and 41 entries of the ITP questionnaire, and it was found that the data fit well with the TPB model: CMIN/DF = 1.092, RMSEA = 0.020, SRMR = 0.037, GFI = 0.886, AGFI = 0.867, NFI = 0.915, CFI = 0.992 (Table 4). The average variance extracted (AVE) and composite reliability (CR) values, which exceeded 0.5 and 0.7 respectively, indicating that the questionnaire demonstrated good convergence validity and internal consistency among the scale items [30] (Table 1). To assess discriminative validity, the square root of the AVE was compared to the correlation coefficients between the five components. The findings revealed that the scale data displayed strong discriminative validity, as the square root of the AVE values were higher than the correlation coefficient between the components [34].

**Table 4:** Fitness index of the model

Fitness Index	CMIN/DF	GFI	AGFI	RMSEA	SRMR	NFI	CFI
**Critical Value**	< 3	> 0.90	> 0.90	< 0.08	< 0.08	> 0.90	> 0.90
**Value of CFA Model**	1.092	0.886	0.867	0.020	0.037	0.915	0.992
**Value of Final Model**	1.431	0.921	0.908	0.032	0.071	0.940	0.981

Note: CMIN/DF (Chi-square Minimum/Degree of Freedom), GFI (Goodness of Fit Index), AGFI (Adjusted Goodness of Fit Index), RMSEA (Root Mean Square Error of Approximation), SRMR (Standardized Root Mean Square Residual), NFI (Normed Fit Index), CFI (Comparative Fit Index)

### Structural equation model

**Fig. 1:**
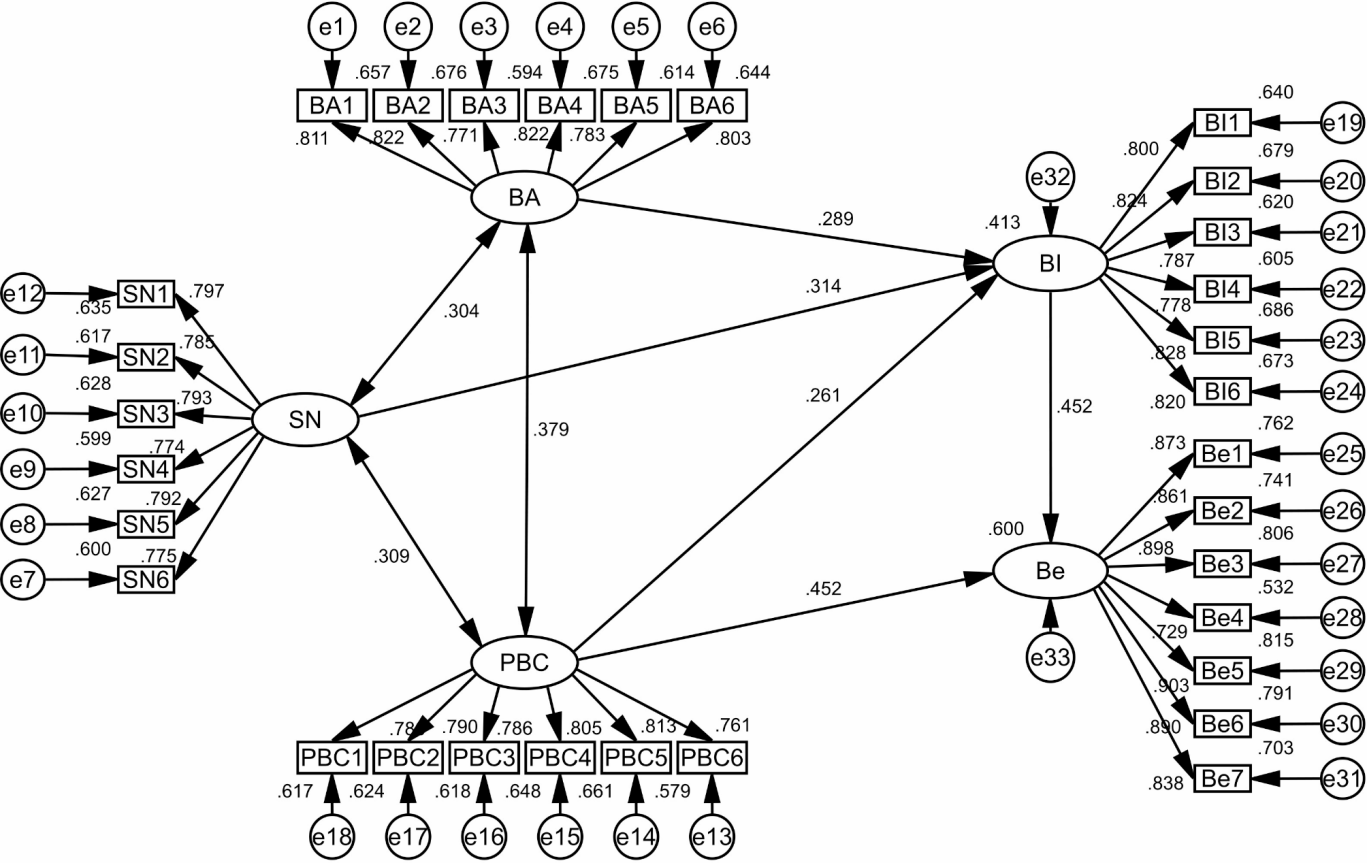
A structural equation model of T2DM patients’ behaviors associated with ITP services based on the theory of planned behavior

The model was rigorously validated through the application of the ML method. Figure 1 illustrates the statistical significance of the path coefficients among all latent variables. It was found that a stronger BI was correlated with a more positive BA (β = 0.289, P < 0.001), greater SN (β = 0.314, P < 0.001), and higher PBC (β = 0.261, P < 0.001). BI (β = 0.452, P < 0.001) strongly influenced behavior associated with ITP services, and the more powerful the BI, the better the behavior’s performance. Furthermore, PBC (β = 0.452, P < 0.001) exerted a similar impact on behavior as BI in terms of direct influence (Table 5). Table 4 presents the results of the goodness-of-fit tests, which confirm the compatibility of the model with the data and the appropriateness of the indicators used in the study.

**Table 5:** The path coefficient among variables

Model Paths			Std.	S.E.	C.R.	P
BA	→	BI	0.289	0.049	5.827	***
SN	→	BI	0.314	0.049	6.516	***
PBC	→	BI	0.261	0.052	5.263	***
BI	→	Be	0.452	0.123	9.947	***
PBC	→	Be	0.452	0.129	9.852	***

Note: ***<0.001

## Discussion

### Main findings

In this study, we evaluated the determinants of behavior associated with ITP services for patients with T2DM. TPB provided acceptable model fit statistics, accounting for 41% of the variance in ITP behavioral intention and 60% of the variance in behavior. This indicated that TPB had sufficient predictive value in explaining behavior associated with ITP services and behavioral intention. The TPB model was found to be in consonance with the survey data, where factors such as BA, SN, and PBC were identified as significant predictors of behavioral intention associated with ITP services. Moreover, both BI and PBC had a direct impact on the actual behavior of patients with T2DM, and their predictive effect was found to be equivalent.

### The influence of BA, SN and PBC on BI

In this study, we observed a significant correlation between the variables of BA, SN and PBC with BI, which was in line with the hypothesis of TPB [23]. The study concluded that the greater the level of BA, the stronger the positive social pressure from the surrounding people, and the more heightened perception and control over the behavioral disorder associated with ITP services, the more likely the participants were to exhibit a willingness to improve their health status through the behavior associated with ITP services. This result was consistent with John Sample’s research that attitude plays a significant role in predicting behavior and BI [35]. Additionally, the study found a significant correlation between patient attitudes and self-care practices [36]. Attitudes and beliefs have been shown to influence disease-related behaviors, resulting in varying levels of diabetes self-management [37]. Higher levels of knowledge and a more positive attitude tend to be associated with better levels of self-management and lower levels of glycosylated hemoglobin [38]. This suggests that the BI associated with ITP services in T2DM patients can be enhanced through health education.

SN generally also affect intentions, but intentions based on attitudes are a better predictor of observed behavior than those based on SN [39]. SN constructs are generally considered to be weak predictors of intention [39], as normative factors have been shown to have low importance as determinants of intention in the behaviors studied. However, in this study, the path coefficient between SN and intentions was found to be the highest among the three antecedents of intentions. This difference may be attributed to the reduced role of attitudes on BI, which is believed to be due to the low education level of patients and the vague concept of SN. Sheeran and Orbell believed that the weak relationship between SN and intention in previous studies is due to the concept of SN not accurately reflecting the influence of society on individual behaviors [40]. The impact of various norm types on a person’s behavioral intention varies, with descriptive norms having been reported to have a higher regression coefficient in predicting intention than SN. This suggests that observing the behaviors of others may be more important in health-related decisions than social pressure from others, and that using peer models could potentially reduce the prevalence of health-risk behaviors and increase the occurrence of healthy behaviors [41, 42]. It may be difficult to manipulate interventions that target particular BA and SN, and the findings suggest that information aimed at increasing the importance of components of health behavior norms (such as promotion of peer pressure and increased family health concerns) should also increase the likelihood of their implementation [43]. The addition of PBC aims to address the phenomenon of incomplete volitional control. Previous research has demonstrated that PBC is a crucial determinant of self-management behavioral intentions in T2DM patients [44]. However, the impact of PBC in this study on the behavioral intentions associated with ITP services in T2DM patients was the least pronounced among the three approaches investigated. This discrepancy may be attributed to the type of behaviors we measured [45]. The study revealed that familiarity acts as a moderating variable between PBC and intentions, with PBC showing a stronger predictive value for familiar behaviors. Notani attributed this stronger relationship to a greater interest in familiar behaviors [46]. As an extension of diabetes self-management behaviors, the behaviors associated with ITP services paid additional attention to patients’ utilization of community health services. Patients’ unfamiliarity with this pattern of service may lead to a lack of interest and weak correlation between PBC and intention. Health education may serve as an effective intervention to reduce this unfamiliarity. Education has a multitude of positive outcomes, several of which exhibit a mutual relationship. For instance, a person who experiences an increased sense of control over their life as a result of their educational pursuits is more likely to adopt healthier habits, which in turn leads to enhanced functionality and a heightened perception of control [47].

### Direct effects of BI and PBC on behavior

Most research applying the TPB does not delve into actual behavior, but instead focuses solely on examining intention [48, 49]. In the model, intention, as a proximal factor affecting behavioral decision-making, has a significant direct impact on behavior [50]. Two studies conducted in Australia reported a strong correlation between intention and behavior [51, 52]. Although TPB is capable of explaining part of behavioral variance, most behavioral variance remains unexplained. In terms of explaining the power of the model on behavior, the study revealed a wide range of R [2] values, ranging from 0.0036 [53] to 0.84 [54], for the explanatory power of intention and PBC on behavior. Despite having similar intention and PBC, individuals often exhibit different behaviors in real situations. To explore the underlying reasons, the concept of implement intention was introduced as a possible cognitive mechanism [55]. Some researchers believe that the occurrence of behavior goes through two stages: the motivation stage and the execution stage. The motivation stage involves the formation of overall intention, which is influenced by attitude, SN and PBC, similar to Ajzen’s concept of BI. The execution stage, which is situated between intention and behavior and involves willingness, is where individuals choose relevant actions that can be implemented by making specific behavior plans. Making specific behavior plans is a typical feature of this stage and also the first step in implementing behaviors [56]. Numerous studies have found that executive intention can promote the occurrence of general behaviors in daily life, such as increasing physical exercise [57] or adopting a healthy diet [58]. For individuals with cognitive impairments, such as those suffering from social function withdrawal, the role of executive behavioral intention in promoting behavior even more noticeable.

Unlike the other two predictors of intention (BA and SN), PBC has been proposed to directly predict behavior, as supported by our findings. Sylvmarie Gatt says PBC is the most predictive factor in regards to actual self-care behavior, with high PBC being reported by participants in associated with taking medication [59]. Additionally, PBC also had a significant direct effect on compliance behavior, that is, adherence to medical protocols was directly explained the extent to the participants’ level of control over their diabetes-related self-care behaviors, while attitudes and SN did not have such an effect [60]. In recent years, studies have found that PBC mainly depends on self-efficacy and control [61]. Self-efficacy has been found to be a significantly predictor of self-management behavior for certain health behaviors, with patients who possess a higher sense of self-efficacy in following diet restrictions, exercising, blood glucose monitoring, and taking care of their feet being more likely to exhibit self-management behavior [18]. Previous studies have also reported a positive correlation between health literacy level and PBC, and identified health literacy as a predictor of PBC [62]. Health education initiatives may focus on promoting health literacy in order to improve self-efficacy, improve PBC, and enhance patients’ awareness of disease controllability and the possibility of behavior change [63, 64].

## Implication

There is great value in both theoretical and practical aspects of this study. First, it could establish a robust and objective theoretical and empirical basis for the subsequent personalized intervention research on T2DM in the community. The discussion of the influencing factors and mechanism of T2DM patients’ behavior associated with ITP services provided a reference for promoting ITP services in a more accurate and efficient manner. Second, it offers valuable insights into optimizing the community’s ITP services for T2DM patients. Behavior associated with ITP services of T2DM patients directly reflected the quality of ITP services in the community. Given the current context of the construction of ITP service systems for chronic diseases in primary healthcare centers, this study delves into the crucial factors and internal mechanisms that restrict the actual behavior of patients with T2DM in the community, which was conducive to develop targeted optimization strategies, improve the formalization and invalidation of ITP services, and expand a new path of ITP service model with Chinese characteristics.

## Limitation

It is necessary to consider the limitations of this study. Firstly, the fact that recruitment of T2DM patients was conducted in one single community health center raises an issue about the generalizability of our findings. To improve sample representation, future studies should consider enrolling patients from a wider range of healthcare facilities, such as hospitals, and clinics across the city. Additionally, the cross-sectional nature of the study limits the ability to establish a definitive causal relationship between beliefs and behavior associated with ITP services, highlighting the need for future longitudinal prospective studies to show causality. Finally, the possibility of recall bias cannot be ruled out in self-reported questionnaires, and it is possible that self-reported intention may be affected by socially expected responses. However, this study undoubtedly provides new insights into this topic and confirms previous research.

## Conclusion

The TPB model was effective in explaining behaviors and intention associated with ITP services. Based on TPB, this study conducted an in-depth study on factors related to intention and behaviors associated with ITP services in T2DM patients. It was found that SN was the strongest predictor of improving behavioral intention, followed by BA and PBC. In T2DM patients, PBC and BI were major predictors of behavior associated with ITP services. Recent studies on the ITP services mainly focus on the current situation and service framework, but systematic research on the behaviors associated with ITP service is still lacking. This study, from the perspective of the demand-side, conducted a comprehensive assessment of the behaviors and influencing factors associated with the ITP services for T2DM patients in the community, which highlights the importance of SN for behavioral change associated with ITP services in patients with T2DM, and provides new ideas and theoretical basis for the optimization of health management in patients with T2DM. In addition, from a practical point of view, T2DM patients often fall into the inertia of “focusing on treatment rather than prevention”, while the ITP services in the community has not yet been fully utilized. Hopefully, this study can provide scientific references for promoting ITP services in the community, and also can help guide T2DM patients’ behaviors associated with ITP services to improve their self-management ability, delay the progression of diabetes, improve patients’ quality of life and achieve better health outcomes.

### Electronic supplementary material

Below is the link to the electronic supplementary material.

Supplementary Material 1

## Data Availability

The datasets analyzed during the current study are available from the corresponding author on reasonable request.
