# Supplementary material for: Factors affecting T2DM patients’ behaviors associated with integrated treatment and prevention services in China
Source: Int J Equity Health. 2023 Oct 19;22:223. doi: 10.1186/s12939-023-02028-9 (PMC10588159; doi:10.1186/s12939-023-02028-9)
Supplement: Supplementary file 1 — Supplementary Material 1 [file 12939_2023_2028_MOESM1_ESM.docx]

**Questionnaire no.**

**Questionnaire of integrated treatment and prevention services for patients with type 2 diabetes**

Dear Sir/Madam,

Hello! First of all, thank you very much for participating in this study! We are conducting this survey to learn about your understanding and behavior towards integrated treatment and prevention (ITP) for type 2 diabetes mellitus (T2DM), so that we can provide better ITP services in the future. It will take up to 15 minutes of your time. To preserve the confidentiality of the information you provide, we will use an anonymous survey. There is no correct or incorrect answer. The most important thing is to express your actual feelings. This survey is only for research purposes. Your meticulous completion of this questionnaire is the most important source of support for our research. Thank you for your patience and support! Let us all work together to ensure a healthy tomorrow!

Diabetes Health Management Research Group, Nanjing Medical University

| **Part I. Basic information of patients** | | |
| --- | --- | --- |
| A1. Your year of birth: | ________(subject to ID card registration) |  |
| A2. Your gender: | 🞎Men 🞎Women |  |
| A3. Your height: | ____ cm |  |
| A4. Your weight: | ____ kg |  |
| A5. Your education level: | 🞎 University and above  🞎 High school/secondary school  🞎 Junior high school  🞎 Primary school  🞎 Illiteracy |  |
| A6. Your marital status: | 🞎 In marriage 🞎 Single |  |
| A7. Your occupation: | 🞎 In employment 🞎 Retired |  |
| A8. Your average monthly income: | 🞎 Less than 3000 Yuan  🞎 3000 (inclusive) to 5000 yuan  🞎 5000 (inclusive) to 8000 Yuan  🞎 8000 (inclusive) to 10000 yuan  🞎 10000 yuan or above |  |
| A9. Type of health insurance you are enrolled in (multiple options are available): | 🞎 State medicine  🞎 Urban employee basic medical insurance  🞎 Commercial insurance  🞎 Urban-Rural Resident Basic Medical Insurance  🞎 Other medical insurance  🞎 No insurance |  |
| A10. Do you have a family history of diabetes? | 🞎Yes 🞎No |  |
| A11. Your diabetes course: | ______years or ______months |  |
| A12. Your diabetes treatment modality: | 🞎 Diet and exercise  🞎 Oral medication only  🞎 Insulin only  🞎 Oral medication combined with insulin  🞎 Other |  |

| **Part Ⅱ. Questionnaire associated with ITP services** | | | | | | | | | | | | | | | | | | | | | |
| --- | --- | --- | --- | --- | --- | --- | --- | --- | --- | --- | --- | --- | --- | --- | --- | --- | --- | --- | --- | --- | --- |
| **Attitude sub-questionnaire** | | | | | | | | | | | | | | | | | | | | | |
|  | Entry | Very important=5 | | Important=4 | | | General=3 | | | | | Unimportant=2 | | | | | Very unimportant=1 | | | | |
| BA1 | Do you think it is important for diabetics to take medication as prescribed by their doctor for blood sugar control? |  | |  | | |  | | | | |  | | | | |  | | | | |
| BA2 | Do you think it is important to cooperate with your doctor on diet control? |  | |  | | |  | | | | |  | | | | |  | | | | |
| BA3 | Do you think it is important to exercise under the guidance of a doctor for blood sugar control? |  | |  | | |  | | | | |  | | | | |  | | | | |
| BA4 | Do you think it is important to monitor blood sugar to adjust treatment and control blood sugar? |  | |  | | |  | | | | |  | | | | |  | | | | |
| BA5 | Do you think it is important to receive diabetes health education for effective diabetes control? |  | |  | | |  | | | | |  | | | | |  | | | | |
| BA6 | Do you think it is important for people with diabetes to receive regular follow-up from healthcare professionals to control their blood sugar? |  | |  | | |  | | | | |  | | | | |  | | | | |
| **Subjective norm sub-questionnaire** | | | | | | | | | | | | | | | | | | | | | |
|  | Entry | Very big =5 | | | Big=4 | | | | General=3 | | | | small=2 | | | | Very small=1 | | | | |
| SN1 | The influence of the surrounding populations on your adherence to the principles of diabetes medication |  | | |  | | | |  | | | |  | | | |  | | | | |
| SN2 | The influence of the surrounding populations on your adherence to the dietary principles of diabetes |  | | |  | | | |  | | | |  | | | |  | | | | |
| SN3 | The influence of surrounding populations on your adherence to the exercise principles for diabetes |  | | |  | | | |  | | | |  | | | |  | | | | |
| SN4 | The influence of surrounding populations on your compliance with blood glucose monitoring for diabetes |  | | |  | | | |  | | | |  | | | |  | | | | |
| SN5 | The influence of surrounding populations on your compliance with health education |  | | |  | | | |  | | | |  | | | |  | | | | |
| SN6 | The influence of surrounding populations on your compliance with regular follow-up? |  | | |  | | | |  | | | |  | | | |  | | | | |
| **Perceived behavioral control sub-questionnaire** | | | | | | | | | | | | | | | | | | | | | |
|  | Entry | Very easy=5 | | Easy=4 | | | | | | common=3 | | | | | Difficult=2 | | | Very difficult=1 | | | |
| PBC1 | For you, the act of taking your medication or (and) insulin injections as prescribed by your doctor |  | |  | | | | | |  | | | | |  | | |  | | | |
| PBC2 | For you, the act of following the principles of the diabetic diet |  | |  | | | | | |  | | | | |  | | |  | | | |
| PBC3 | For you, the act of exercising regularly under the guidance of a doctor |  | |  | | | | | |  | | | | |  | | |  | | | |
| PBC4 | For you, the act of regular self-glucose monitoring |  | |  | | | | | |  | | | | |  | | |  | | | |
| PBC5 | For you, regular diabetes health education behavior |  | |  | | | | | |  | | | | |  | | |  | | | |
| PBC6 | For you, the act of receiving regular follow-up |  | |  | | | | | |  | | | | |  | | |  | | | |
| **Behavioral intention sub-questionnaire** | | | | | | | | | | | | | | | | | | | | | |
|  | Entry | | Strongly agree=5 | | | agree=4 | | | | | General=3 | | | | | disagree=2 | | | Strongly disagree=1 | | |
| BI1 | Do you plan to take your medication or/and insulin shots as prescribed by your doctor? | |  | | |  | | | | |  | | | | |  | | |  | | |
| BI2 | Do you plan to follow the Diabetes diet? | |  | | |  | | | | |  | | | | |  | | |  | | |
| BI3 | Do you plan to exercise regularly? | |  | | |  | | | | |  | | | | |  | | |  | | |
| BI4 | Do you plan to self-monitor your blood sugar regularly? | |  | | |  | | | | |  | | | | |  | | |  | | |
| BI5 | Do you intend to receive regular diabetes health education? | |  | | |  | | | | |  | | | | |  | | |  | | |
| BI6 | Do you intend to receive regular follow-up from your health care provider? | |  | | |  | | | | |  | | | | |  | | |  | | |
| **Behavior sub-questionnaire** | | | | | | | | | | | | | | | | | | | | | |
|  | Entry | Always=5 | | Often=4 | | | | Sometimes=3 | | | | | | Seldom=2 | | | | | | Never=1 |  |
| AB1 | During the past six months, have you used a combination of diet, exercise and medication to control your blood sugar? |  | |  | | | |  | | | | | |  | | | | | |  |  |
| AB2 | During the past six months, have you controlled the amount of food you eat per day as recommended by your doctor or dietitian? |  | |  | | | |  | | | | | |  | | | | | |  |  |
| AB3 | During the past six months, have you been on a low-fat, oil-free diet as recommended by your doctor or dietitian? |  | |  | | | |  | | | | | |  | | | | | |  |  |
| AB4 | During the past six months, have you been doing moderate-intensity exercise such as brisk walking or jogging for more than 30 minutes on more than 5 days per week? |  | |  | | | |  | | | | | |  | | | | | |  |  |
| AB5 | During the past six months, do you carry candy with you in case of hypoglycemia when you exercise? |  | |  | | | |  | | | | | |  | | | | | |  |  |
| AB6 | During the past six months, have you been wearing the right size shoes and socks? |  | |  | | | |  | | | | | |  | | | | | |  |  |
| AB7 | During the past six months, have you checked your feet for blisters, chapped skin, chafing, etc.? |  | |  | | | |  | | | | | |  | | | | | |  |  |
| AB8 | Have you visited a medical institution regularly for foot check-ups over the past year? |  | |  | | | |  | | | | | |  | | | | | |  |  |
| AB9 | During the past six months, have you taken your medication on time and in accordance with your doctor's instructions? |  | |  | | | |  | | | | | |  | | | | | |  |  |
| AB10 | During the past six months, have you measured your blood sugar as often as your doctor ordered? |  | |  | | | |  | | | | | |  | | | | | |  |  |
| AB11 | Do you follow your doctor's advice to check your HbA1c every 3 to 6 months? |  | |  | | | |  | | | | | |  | | | | | |  |  |
| AB12 | During the past six months, have you had your blood pressure measured regularly as ordered by your doctor? |  | |  | | | |  | | | | | |  | | | | | |  |  |
| AB13 | During the past year, have you visited a medical institution regularly to check your blood lipids? |  | |  | | | |  | | | | | |  | | | | | |  |  |
| AB14 | During the past year, have you visited a medical institution regularly to check your kidney function? |  | |  | | | |  | | | | | |  | | | | | |  |  |
| AB15 | During the past year, have you visited a medical institution regularly for eye examination? |  | |  | | | |  | | | | | |  | | | | | |  |  |
| AB16 | Do you have a face-to-face follow-up at least once every 3 months? |  | |  | | | |  | | | | | |  | | | | | |  |  |
| AB17 | Do you attend at least one health seminar every month? |  | |  | | | |  | | | | | |  | | | | | |  |  |
